# The Role of Gut Microbiome in the Pathogenesis and the Treatment of Inflammatory Bowel Diseases

**DOI:** 10.7759/cureus.54569

**Published:** 2024-02-20

**Authors:** Andrea Vidal-Gallardo, Juan E Méndez Benítez, Leticia Flores Rios, Luis F Ochoa Meza, Rodrigo A Mata Pérez, Edgar Martínez Romero, Andres M Vargas Beltran, Jose L Beltran Hernandez, Douglas Banegas, Brenda Perez, Marily Martinez Ramirez

**Affiliations:** 1 Surgery, Universidad de los Andes, Merida, VEN; 2 Internal Medicine, Instituto Salvadoreño del Seguro Social, San Salvador, SLV; 3 General Medicine, Universidad Autónoma de Ciudad Juárez, Ciudad Juarez, MEX; 4 General Surgery, Hospital General ISSSTE Presidente General Lázaro Cárdenas, Chihuahua, MEX; 5 General Practice, Facultad de Estudios Superiores Iztacala, Universidad Nacional Autónoma de México, Ciudad de México, MEX; 6 General Practice, Benemérita Universidad Autónoma de Puebla, Puebla, MEX; 7 General Practice, Benemerita Universidad Autonoma de Puebla, Puebla, MEX; 8 General Practice, Centro de Estudios Universitarios Xochicalco, Mexicali, MEX; 9 General Medicine, Universidad Nacional Autonoma de Honduras, San Pedro Sula, HND; 10 Nutrition, Universidad ICEL, Ciudad de México, MEX; 11 Internal Medicine, Universidad Nacional Autonoma de México, Mexico City, MEX

**Keywords:** treatment strategies, ibd, inflammatory bowel disease, immune dysregulation, gut microbiome

## Abstract

Inflammatory bowel disease (IBD), which includes ulcerative colitis and Crohn's disease, is a chronic condition characterized by inflammation of the gastrointestinal tract. Its exact cause is unknown, but it's thought to result from a dysregulated immune response influenced by various factors, including changes in the intestinal microbiota, diet, lifestyle, and genetics. The gut microbiome, consisting of diverse microorganisms, plays a crucial role in maintaining physiological balance, with its disruption leading to inflammatory responses typical of IBD. Treatments primarily aim at symptom control, employing immunomodulators, corticosteroids, and newer approaches like probiotics, prebiotics, fecal transplants, and dietary modifications, all focusing on leveraging the microbiota's potential in disease management. These strategies aim to restore the delicate balance of the gut microbiome, typically altered in IBD, marked by a decrease in beneficial bacteria and an increase in harmful pathogens. This review underscores the importance of the gut microbiome in the pathogenesis and treatment of IBD, highlighting the shift towards personalized medicine and the necessity for further research in understanding the complex interactions between the gut microbiota, immune system, and genetics in IBD. It points to the potential of emerging treatments and the importance of a multifaceted approach in managing this complex and challenging disease.

## Introduction and background

Inflammatory bowel disease (IBD) encompasses a group of chronic, idiopathic, and multifactorial disorders, primarily ulcerative colitis (UC) and Crohn's disease (CD). These conditions are characterized by inflammation of the gastrointestinal tract. The incidence of IBD varies globally, being higher in Westernized countries such as North America (up to 23.14 per 100,000 people) and Europe (up to 57.9 per 100,000 people). In contrast, the incidence in Asian and Middle Eastern countries reaches 6.5 per 100,000 people. Notably, since the latter half of the 20th century, there has been an increase in IBD incidence in countries in Asia, South America, and Africa. This trend correlates with the Westernization of these regions, significantly impacting patients' quality of life and imposing considerable costs on healthcare systems [1-3].

The pathophysiology of IBD is complex and not fully understood. However, the prevailing theory suggests that a dysregulated immune response plays a central role, influenced by various factors including changes in the intestinal microbiota. Studies have demonstrated that both external factors, such as diet and lifestyle, and endogenous factors, including the microbiota, immunological elements, and genetic expression alterations, contribute to the development of the pathology [4,5]. Research involving animal models and human studies has established a link between the microbiota and the development, progression, and response to treatment in IBD [6,7].

The microbiota, comprising organisms such as bacteria, viruses, protozoa, and fungi that inhabit and interact with the human body [8], is most concentrated in the intestinal tract. This concentration varies within the body, being lower in the stomach and progressively increasing along the digestive tract, with the highest quantity found in the colon [9]. Comparisons between the microbiota of individuals with IBD and those without it reveal significant compositional differences. However, due to technical and methodological challenges in studies, it remains difficult to ascertain whether these differences are a cause or consequence of IBD [8].

IBD, an incurable disease, primarily focuses on symptom control and prevention of disease flares. The disease is associated with the occurrence of gastrointestinal neoplasms. Current treatment strategies include the use of immunomodulators and corticosteroids. Given the numerous studies highlighting the relationship between intestinal microbiota and IBD, there has been an increase in clinical trials exploring treatments such as probiotics, prebiotics, fecal transplants, and long-term dietary changes [10-14]. This review aims to explore the primary characteristics of IBD, the role of the intestinal microbiota in the natural history of the disease, new therapeutic approaches, and future directions. It offers fresh perspectives on gut-brain-axis interactions and the potential of microbiome-based therapies (MBT), bridging the gap between fundamental microbiome research and clinical practice and advocating for a shift towards personalized medicine in IBD treatment.

## Review

The microbiome: Composition and functions

**Figure 1:**
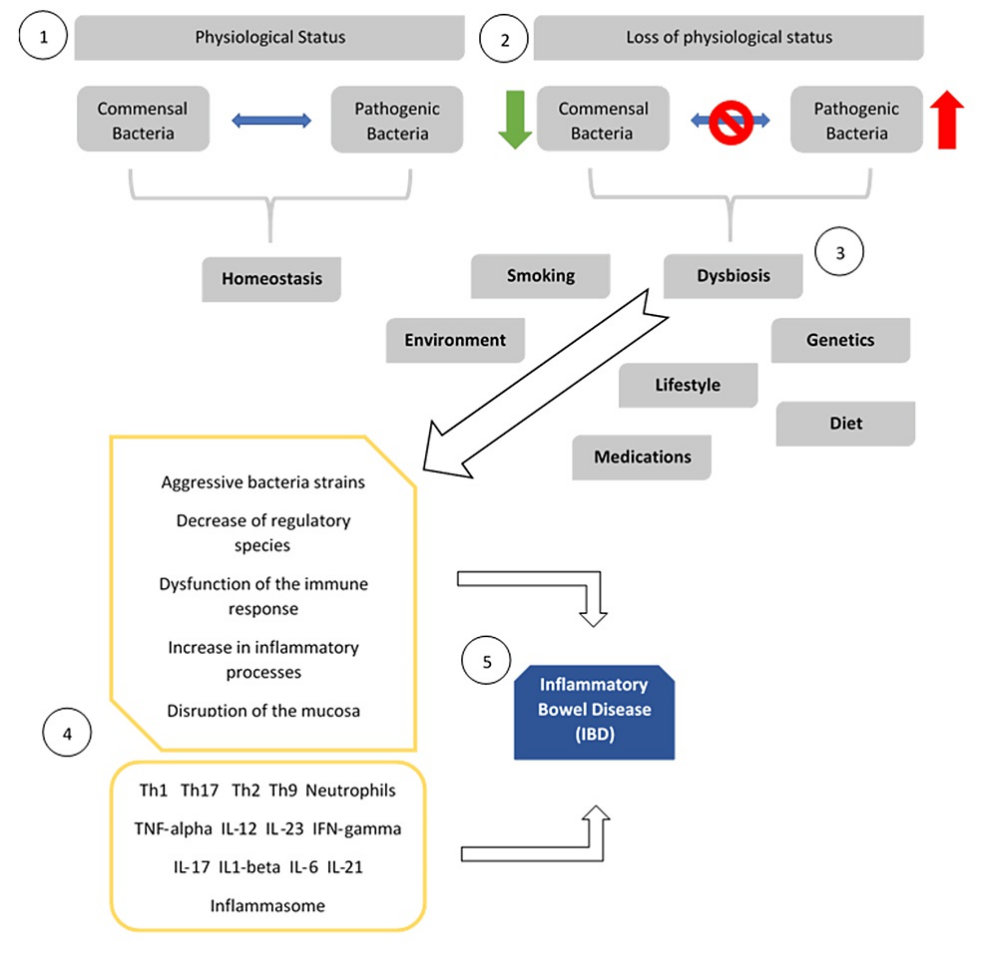
Dysbiosis as a main trigger for IBD 1 - Physiological homeostasis. 2 - Disruption of homeostasis leads to a state of "intestinal dysbiosis. 3 - Dysbiosis combined with various factors triggers inflammatory signaling mechanisms. 4 - Leading to inadequate immune responses and the production of cytokines and inflammatory mediators. 5 - The resultant persistent inflammatory environment is a hallmark of IBD [22]. IBD: Inflammatory bowel disease Image Credits: Luis Fernando Ochoa Meza

The human colon harbors a highly diverse and complex microbiome. The intestinal microbiota is estimated to contain approximately 100 billion bacteria, encompassing more than 1,000 taxa (including species, genera, and families), all playing a pivotal role in maintaining homeostasis [15]. Recent years have seen a surge in interest in identifying and quantifying human-resident bacteria [16]. Predominantly, the human microbiome comprises five main bacterial phyla: *Firmicutes*, *Bacteroidetes*, *Proteobacteria*, *Actinobacteria*, and *Verrucomicrobia *[15]. Its composition is influenced by a myriad of environmental factors such as diet, drugs/antibiotics, lifestyle, and host genetic components [17-20], exemplified by the correlation between host mitochondrial DNA haplogroups and the microbiome structure (Figure 1) [17,21].

Under normal physiological conditions, intestinal bacteria and their human host coexist in equilibrium. The digestion of carbohydrates requires specific enzymes, primarily glycoside hydrolases (GHs). Humans produce only 17 types of these enzymes, while certain bacteria, such as the abundant *Bacteroides*, encode 260 GHs, thus enabling the host to metabolize carbohydrates [16]. In the colon, the microbiota primarily ferments natural non-starch polysaccharides to produce short-chain fatty acids (SCFAs) like acetate, butyrate, and propionate. These SCFAs exert immunomodulatory and anti-inflammatory effects, including modulating intestinal barrier permeability, reducing oxidative stress, and diminishing inflammation through inhibiting mediators such as NF-κB and IL-8 and affecting cellular communication [2,23]. However, disruption of homeostasis leads to a state of "intestinal dysbiosis," characterized by an imbalance between beneficial and harmful bacteria. This dysbiosis, combined with various factors, triggers inflammatory signaling mechanisms, leading to inadequate immune responses and the production of cytokines and inflammatory mediators. The resultant persistent inflammatory environment is a hallmark of IBD (as illustrated in Figure 1). Variations in this environment can be monitored using non-invasive biomarkers such as fecal tryptic activity, oxidative response, or lipid and glycan metabolism pathways [9].

In IBD, a restriction of biodiversity and an imbalanced bacterial composition have been demonstrated. There is a reduction in the abundance of beneficial bacteria such as *Clostridium groups IV *and *XIVa*, *Sutterella*, *Roseburia*, and *Faecalibacterium prauznitzii*. Conversely, an increase in pathogens like *Proteobacteria *members (including *Escherichia*, *Salmonella*, *Yersinia*, *Helicobacter*, or *Vibrio*), *Veillonellaceae*, *Pasteurellaceae*, *Fusobacterium *species, and *Ruminococcus gnavus *has been extensively reported in IBD patients [9,24,25]. Specific studies have noted a decrease in *Roseburia *spp. and a significant increase in *R. gnavu*s, contributing to impaired barrier stability and inflammation [25,26].

IBDs: A clinical perspective

IBD encompasses two primary forms: CD and UC, both chronic idiopathic inflammatory conditions. Differentiating between these diseases relies on clinical, endoscopic, histologic, and radiologic features. The pathogenesis of IBD is not fully understood, yet there is substantial evidence suggesting it results from a complex interplay of microbiome and gut dysbiosis, genetic susceptibility, and environmental factors such as diet, drugs, infections, geography, and stress [27-30].

The predominant symptoms of CD include abdominal pain, diarrhea, weight loss, and fever. Less common symptoms are bloody stool, bleeding, and constipation. UC's major symptoms are diarrhea, abdominal pain, and bloody stool, with mucus, weight loss, occasional fever, anemia, and constipation being less frequent [31]. Table 1 summarizes CD and UC's clinical, endoscopic, and histological characteristics. Notably, both diseases are associated with extraintestinal manifestations, observed in 19.9% of CD and 10.9% of UC cases [31]. Some of the characteristics the clinical needs to be aware of are inflammatory markers and nutritional status, as these patients can have an elevation of liver enzyme levels and vitamin deficiencies that need to be treated [32-35]. Some patients usually opt to use herbal products such as silymarin, which has been shown to reduce some markers, such as liver enzymes, and to regulate the microbiome [36,37].

**Table 1:** Characteristics of Crohn’s disease and ulcerative colitis [31,38,39]

Feature	Crohn’s Disease	Ulcerative Colitis
Predominant Symptom	Abdominal Pain	Diarrhea
Sex Ratio	1.65	1.28
Disease Location	Mouth to Anus	Limited to Colon
Disease Pattern	Patchy	Continuous
Endoscopic Appearance	Aphthoid lesions/ulcers forming longitudinal ulcers with a cobblestone appearance	Mild: absence of vascular pattern, fine granular mucosa, erythema; Moderate: coarse mucosa, erosions, small ulcers, contact bleeding; Severe: widespread ulcers, marked spontaneous bleeding
Histology	Transmural inflammation and granulomas	Change limited to mucosa and submucosa

IBD is now recognized as a global disease linked to significant morbidity, mortality, and healthcare costs [1]. Although IBD is a chronic disease without a definitive cure, effective treatments are available. Currently, management for IBD is characterized by a multifaceted strategy that encompasses interventions from lifestyle modifications (specific diets, stress management, regular exercise), pharmacological therapy (corticoids, immunomodulators), biological therapies (anti-TNF agents, anti-integrins, anti-IL-12/23) and constant surveillance and monitoring of the patient. IBD significantly impacts patients' quality of life and social functioning, both physically and emotionally. For example, muscle atrophy is common among IBD patients, with 42% experiencing myopenia, 34% pre-sarcopenia, and 17% sarcopenia, leading to increased risks of therapy failure, postoperative complications, and low body mass index [40]. Additionally, the prevalence of depression (25.2%) and anxiety (32.1%) among IBD patients further exacerbates the burden on healthcare systems [41].

Microbiome alterations and role in IBD pathogenesis

IBD is characterized by a reduced diversity of gut microbiota, with a significant decrease in *Firmicutes *and an increase in *Bacteroidetes*, leading to an imbalance between pathogenic and commensal microorganisms [42-44]. This alteration stems from a combination of genetic predisposition and environmental exposures, cumulatively disrupting the gut microbiome's composition and diversity, which in turn leads to pathological immune activation and a pro-inflammatory state [45].

In IBD, gut microbiota contributes to aberrant immune activation, where microbial accumulation and local penetration during early dysbiosis initiate a proinflammatory phase [46]. This is characterized by inadequate immunosuppressive control by T-regulatory cells, resulting in an abnormal T-cell response and tissue damage in the intestinal mucosa [47]. Dysbiosis in IBD impairs vital immunomodulatory functions and disrupts the intestinal barrier's integrity, increasing its permeability and facilitating inflammation [48]. Studies have shown disruptions in the gut microbiome in IBD due to host or environmental factors, leading to decreased species diversity, particularly in the *Firmicutes *phylum in patients with CD compared to healthy controls [49-52].

These alterations are linked with neuropsychiatric/neurological disorders via the "Gut-Brain-Axis," indicating a bidirectional relationship between the gastrointestinal and nervous systems [53]. This is further supported by next-generation sequencing, which reveals characteristic microbiome alterations in IBD patients, including decreased levels of beneficial bacteria and increased levels of pathogens [9]. The role of fungi and viruses in the gut microbiome is increasingly recognized in the context of IBD. Studies have specifically linked the presence of *Candida albicans* with CD, indicating a broader spectrum of microbial involvement in IBD pathogenesis beyond bacteria [43,54]. This aspect underscores the complexity of dysbiosis in IBD and its multifactorial nature in influencing the disease's progression and severity. The "Gut-Brain-Axis" (MGB Axis) illustrates a significant link between IBD and neuropsychiatric/neurological disorders, where gut microbiome dysbiosis affects the integrity of both intestinal and blood-brain barriers [53,55]. The MGB Axis involves humoral, neural, immune, and endocrine systems, with microbiome dysbiosis in IBD triggering cytokine release and immunomodulatory responses, affecting both gastrointestinal and nervous systems. Bidirectional relay of inflammatory signals between the intestine and the CNS occurs through three main pathways: Systemic-humoral pathway - systemic release of gut-derived inflammatory factors can alter BBB integrity and lead to defects in brain development; Immune pathway - activated enteric immune cells can translocate to the CNS to promote or inhibit neuroinflammation. Stress responses to CNS insults can alter the gut microbiome and stimulate inflammatory immune cells, which then migrate to the CNS and aggravate neuroinflammation; Neuronal pathway - stimulation of inflammation-sensing afferent vagal and dorsal root ganglion (DRG) neurons triggers CNS neural circuits involved in hypothalamic-pituitary-adrenal (HPA) activation, sickness behavior, and visceral pain perception [56].

Microbiome profile differences in active and inactive stages of IBD have been observed, with increases in *F. prausnitzii *and *Clostridiales *in inactive IBD, and *Proteobacteria *in active IBD [9]. Animal models have advanced the understanding of IBD's immunological and histopathological features, aiding in developing novel therapeutic approaches [57]. These findings highlight the dynamic nature of the microbiome in IBD and its potential as a target for treatment strategies.

Therapeutic implications and interventions

IBD treatment involves interventions targeting the gut microbiota, with probiotics playing a crucial role. Probiotics, defined by WHO/FAO as living microorganisms conferring health benefits, act through mechanisms like blocking adhesion sites and producing anti-inflammatory substances, showing potential in effectively managing IBD [58]. Their impact on cytokine production, regulatory T-cell induction, and microbial killing highlights their therapeutic potential [58].

In contrast, fecal microbiota transplantation (FMT) and prebiotics contribute to IBD improvement by selectively stimulating beneficial bacteria growth in the colon. While both approaches aim to establish a healthy microbial balance, prebiotics achieve this by modulating the existing endogenous microflora. These interventions represent promising avenues for managing IBD, addressing the dysregulation of gut microbiota associated with the disease. Multiple clinical trials evaluating probiotic strains such as *Lactobacillus*, *Bifidobacterium*, and *Escherichia coli.* Nissle (1917) indicates their emerging role in inducing remission and maintaining health in UC patients [59-61]. However, variations in results among trials suggest that probiotic effectiveness depends on factors like strain specificity, dosage, and delivery method (Table 2) [61], emphasizing the need for further research and consideration of these variables in clinical applications.

**Table 2:** Key findings from clinical trials evaluating microbiome-targeted interventions in IBD IBD: Inflammatory bowel disease, RCT: Randomized controlled trials, FCAL: Fecal calprotectin, US: Ulcerative colitis, CD: Crohn's disease, CRP: C-reactive protein, FMT: Fecal microbiota transplantation

Author	Country	Number of Individuals	Type of Study	Intervention	Result	Conclusion
Bjarnason et al., 2019 [62]	UK	167	RCT	Probiotic supplementation	FCAL score significantly reduced in UC patients but not in CD patients taking probiotics compared to placebo.	This multi-strain probiotic can reduce inflammation in UC but not in CD.
Zocco et al., 2006 [63]	Italy	187	RCT	Lactobacillus GG (Probiotic)	Lactobacillus GG prolongs relapse-free time	Probiotic maintains ulcerative colitis remission.
Lindsay et al., 2006 [64]	UK	10	RCT	15 g/day fructooligosaccharide (prebiotic)	Reduced disease activity vs. placebo	Prebiotics may be beneficial in active Crohn's disease.
Fachinn et al., 2020 [65]	Italy	49	RCT	Oral administration of microencapsulated sodium butyrate (Prebiotic)	Microbiota changed in IBD patient	Sodium butyrate supplementation promotes the growth of bacteria with potential anti-inflammatory action.
Ojetti et al., 2022 [66]	Italy	119	RCT	*Limosilactobacillus reuteri* (Probiotic) for 10 days	Inflammatory markers (C-RP value and calprotectin level) were reduced in the probiotic group in contrast to the placebo group.	Probiotic supplementation effectively reduces inflammation in IBD.
Moayyedi et al., 2015 [67]	Canada	75	RCT	FMT	24% remission with FMT vs. 5% placebo	FMT induces remission in UC.
Paramsothy et al., 2017 [68]	Australia	85	RCT	FMT	27% remission with FMT vs. 8% placebo	FMT is effective for inducing UC remission.
Zhang et al., 2020 [69]	China	970	RCT	FMT by washed preparation vs manual FMT	7/9 patients achieved positive clinical response	FMT by washed preparation is an effective intervention for active UC and CD.

Regarding CD, strong evidence has shown that high doses of probiotics and prebiotics used in active CD improve clinical symptoms and promote a reduction or remission in the use of prednisolone [70]. The evidence and interpretation of the results are considered precise enough, although the number of available clinical trials and observational studies is very limited [71].

The International Scientific Association for Probiotics and Prebiotics (ISAPP) revised the prebiotic definition to include any host-used substrate for health benefits [72]. Studies show that fructo-oligosaccharides (FOS) in prebiotics promote beneficial bifidobacteria, modify dendritic cell activity, and reduce CD activity [64]. Larger trials are needed to confirm prebiotic efficacy in maintaining remission.

FMT, a novel IBD therapeutic strategy, aims to restore a healthy microbial balance by introducing a fecal bacterial community from a healthy donor to the recipient. Multiple RCTs explored FMT's utility in both UC and CD (Table 1), showing higher rates of remission and improvement in UC compared to placebo or standard therapy [68,73]. There is strong evidence that shows that found an overall beneficial effect of FMT in UC, sparking significant interest as a promising IBD intervention [74]. While therapeutic microbiome targeting holds promise for modifying IBD outcomes, there's heterogeneity in intervention responses based on disease phenotype, patient genetics, and microbiome composition. Further research should identify predictive response biomarkers for precise gut microbiota manipulation.

Limitations in probiotic treatment for IBD include individual responses influenced by genetic factors, age, and lifestyle. Studies show probiotics are comparable to mesalazine in inducing remission in UC, but lack significant effects in CD. Limited studies and small samples necessitate more extensive research to define probiotics' effectiveness in treating IBD.

In the context of FMT, it is crucial to address safety concerns. Noteworthy among these are potential long-term effects, the risk of infectious agent transmission, the lack of standardized protocols, variations in donor sample preparation, and uncertainties regarding regulatory frameworks. Additionally, the potential for unintended consequences or adverse events associated with FMT necessitates comprehensive research to address these concerns and establish its safety and efficacy.

Future directions

Microbiome research in IBD management has the potential to lead to personalized and targeted therapies. By gaining a deeper understanding of the complex interactions between the gut microbiota, immune system, and genetic factors, researchers may be able to develop more effective treatments [75]. For example, the microbiome-based approach, particularly the use of metabolite-based "postbiotics," offers a promising alternative by targeting downstream signaling pathways of the microbiome and acting to mitigate the negative effects of dysbiosis. This approach has the potential to counteract and correct the negative effects of dysregulation of metabolites involved in host-microbe interactions, providing a more targeted and potentially safer therapeutic option for IBD patients [76].

The economic and resource implications of implementing MBT are multifaceted. On one hand, developing and implementing MBT may require significant investment in research, development, and clinical trials. This can include the costs associated with identifying and characterizing specific microbial strains, conducting preclinical and clinical studies, and obtaining regulatory approvals for new therapies. Additionally, producing and quality control microbiome-based products, such as probiotics or FMT materials, may also entail substantial costs. On the other hand, the successful implementation of MBT can potentially reduce long-term healthcare costs associated with chronic conditions such as IBD. By targeting the underlying causes of these diseases and promoting gut health, MBT may lead to improved disease management, reduced hospitalizations, and lower healthcare utilization in the long term. This could result in cost savings for healthcare systems and enhanced quality of life for patients [77].

Furthermore, advancements in high-throughput technologies for analyzing the microbiome may lead to the development of more integrative analysis methods, enabling the construction of an IBD network and the identification of specific therapeutic targets. Research into probiotics-based interventions in IBD has been dominated by employing species from two major genera (i.e., *Bifidobacteria *and *Lactobacillus*), but technological advancements are expanding and diversifying the bacterial species of interest. In particular, the progressing fields of metagenomics coupled with whole genome sequencing and metabolomics may aid in identifying new immunomodulatory strains and their mucosal health-promoting products for potential implications in clinical IBD. Using engineered bacteria for therapeutic purposes in IBD is a field of recent emergence, and engineered microbes have demonstrated great potential for altering host immune responses in experimental mice models [78,79].

Microbiome-based interventions, such as probiotics, raise ethical and regulatory considerations. Probiotics are often marketed as a remedy for specific conditions, blurring the line between food and medication. This shift in usage necessitates tighter regulations to ensure patients receive the prescribed dose, viable organisms, and products manufactured to the same standards as drugs. One of the key ethical considerations is the need for a consistent and internationally harmonized approval process to ensure maximal health benefits and minimal health risks for consumers. In terms of regulatory frameworks, it is important to address the existing limitations and ambiguities in the approval process for probiotic products. For example, the current regulatory system in the European Union (EU) has limitations such as the lack of clarity in distinguishing between fermented foods, probiotic-based products, and genetically modified organisms (GMOs) derived from plant and animal origin. This highlights the need for a more precise and distinct compilation of the approval process for probiotic-based food products and pharmaceuticals [67,80]. Probiotics need tighter regulations to ensure efficacy, proper dosing, and manufacturing standards, especially as they are increasingly used to treat specific diseases [81].

It is imperative to consider the necessity for healthcare providers who are adequately trained in the utilization of novel therapies and approaches, such as FMT. Furthermore, it is essential to acknowledge the variation in socioeconomic backgrounds of these providers, as not all healthcare professionals may have access to such therapies and the requisite training. This disparity can have significant implications for the implementation and effectiveness of these innovative treatments [82]. In conclusion, we propose undertaking a systematic review of this subject matter to enhance our understanding and provide a more objective perspective. The execution of such a review should follow the recognized steps and standards, ensuring that the findings are robust, replicable, and reflective of the best available evidence [83].

## Conclusions

The gut microbiome is integral to the pathogenesis and treatment of IBD like CD and UC. The complexity of IBD involves a dysregulated immune response influenced by microbiota, genetics, and environment. Studies show marked differences in the gut microbiota of IBD patients compared to healthy individuals, guiding therapeutic strategies. Treatments such as probiotics, prebiotics, fecal transplants, and dietary changes aim to rebalance gut microbiota. Probiotics have shown efficacy in managing UC, while FMT has emerged as a promising therapy, especially for UC.

Despite their potential, MBT face challenges, including economic and resource considerations. Technological advances in metagenomics are leading to more personalized IBD treatments. Ethical and regulatory aspects, particularly in probiotic product classification and quality control, are crucial. Standardized, globally harmonized approval processes are essential for these therapies. Future research should focus on identifying microbial targets, understanding host-microbiota interactions, and developing personalized treatments. The gut-brain axis also presents new research opportunities. The aim is to enhance patient outcomes and quality of life through targeted microbiome interventions.
